# Global Warming Impacts Suitable Habitats of the Subtropical Endemic Tree *Acer pubinerve* Rehder, Newly Recorded in Jiangsu Province, China

**DOI:** 10.3390/plants14131895

**Published:** 2025-06-20

**Authors:** Jie Miao, Xinyu Zhang, Zhi Yang, Chao Tan, Yong Yang

**Affiliations:** Co-Innovation Center for Sustainable Forestry in Southern China, College of Life Sciences, Nanjing Forestry University, Nanjing 210037, China; miaoj@njfu.edu.cn (J.M.); zhangxy0812@njfu.edu.cn (X.Z.); zhiyang@njfu.edu.cn (Z.Y.); tanchao@njfu.edu.cn (C.T.)

**Keywords:** *Acer pubinerve*, global warming, MaxEnt, species modeling, endemic species

## Abstract

Global warming has caused the change of the geographical distribution of many species and threatened the living of species on earth. It is important to describe and predict the response of these species to current and future climate changes to conserve and utilize the endemic forest species. *Acer pubinerve* of the Sapindaceae is an important forest tree species endemic to China, our recent fieldwork recorded *A. pubinerve* in the Jiangsu province for the first time, representing the northernmost known occurrence of the species. In this study, we compiled an occurrence dataset of *A. pubinerve* based on field investigation, herbarium specimen data and literature, and mapped the resource distribution of this endemic forest species in China. Then, we used the optimized MaxEnt model to predict the potential suitable areas of the species under current climate conditions and future climate change scenarios and studied the impacts of environmental variables on the suitable areas of the species. The MaxEnt model, optimized with a regularization multiplier of 0.5 and a feature combination of linear and quadratic terms, exhibited the best predictive performance. The prediction accuracy of the model was extremely high and the AUC values of training and test data were 0.995 and 0.998, respectively. We found that the leading environmental variables affecting the potential distribution of *A. pubinerve* include the mean temperature of warmest quarter, the mean temperature of driest quarter, and the annual precipitation. Under the current climatic condition, the suitable distribution area of *A. pubinerve* is 165.68 × 10^4^ km^2^, mainly located in the provinces of Zhejiang, Fujian, Jiangxi, Hunan, Guangdong, and Guangxi. Compared with the suitable area under the current climate, the total suitable areas of *A. pubinerve* is projected to expand toward the north under the future climate change scenarios SSP126, SSP370, and SSP585, while its center shows a general trend of westward migration. Our study lays the foundation for conservation and resource utilization of this endemic tree species in China.

## 1. Introduction

Global climate change has received international attention [1,2]. The IPCC’s Fifth Assessment Report (2014) pointed out that global temperature would keep rising in the future, and this global climate change will inevitably have profound impacts on the geographical distribution patterns of the species. In this context, the distribution range of plants will undergo significant changes, i.e., expansion or contraction of suitable habitats and shifts of the distribution center [3]. This distributional shift not only threatens the genetic diversity of the species but may also disrupt existing interspecific interaction networks within the ecosystem. Therefore, accurately quantifying climate response mechanisms and predicting the change of future suitable habitats have become core scientific issues in the development of conservation strategies. This approach not only helps to reveal the key environmental factors influencing the living of plant species, but also provides theoretical support for plant resource conservation and cultivation practices [4].

Species distribution models (SDMs) provide powerful tools for assessing the effects of environmental variables on species distributions [5]. Among them, the Maximum Entropy (MaxEnt) model has become a widely applied method due to its unique advantages. Compared with traditional models such as Random Forest (RF) and Generalized Linear Models (GLM), MaxEnt has demonstrated strong robustness under small sample conditions, and its predictive accuracy and explanatory power have been widely recognized in the academic community [6,7]. The Maximum Entropy (MaxEnt) model is an efficient tool for species distribution prediction and has been widely applied in various fields. This model is not only used to assess suitable plantation areas for crops [8,9], but is also used to predict the potential suitable habitats for wild plants [10,11] and animals [12], and evaluate the spreading risk of alien invasive species [13]. It is noteworthy that parameter optimization can significantly improve the performance of the MaxEnt model and prevent overfitting, thereby enabling more accurate simulations of species distribution dynamics under different climate scenarios and providing a scientific basis for species conservation and resource utilization [14,15].

**Figure 1:**
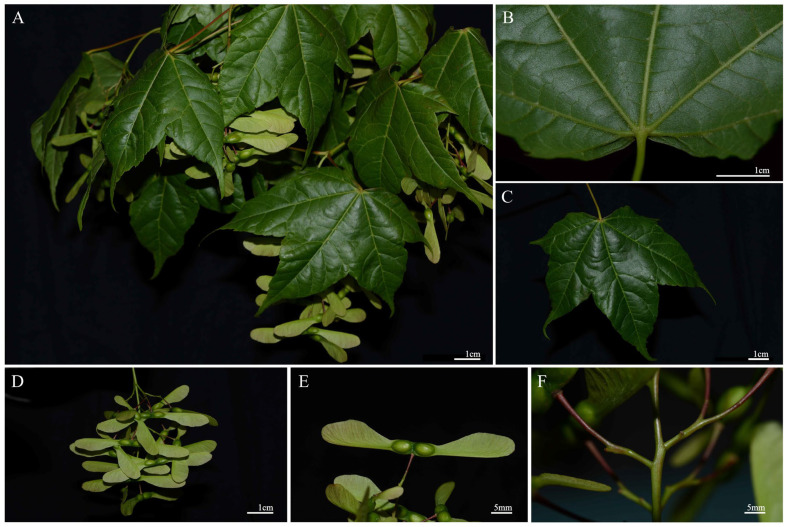
Morphological characters of *Acer pubinerve* of the Sapindaceae. (**A**) Leafy and fruiting branch; (**B**) leaf lower surface; (**C**) leaf upper surface; (**D**) infructescence; (**E**) samara displaying the two horizontal wings; (**F**) infructescence portion displaying the branching pattern. Photograph by Zhi Yang.

*Acer* of the Sapindaceae is primarily distributed across the northern temperate zone. Some species of the genus are well-known for their highly ornamental value, e.g., *Acer buergerianum*, *A. pictum* subsp. *mono*, and *A. wilsonii*, while some others are used for different purposes, i.e., maple syrup of *A. saccharum* and timber for furniture [16,17,18]. *Acer pubinerve* is a subtropical tree species endemic to China. It is a deciduous tree, is 7–10 m tall, dark gray, with smooth bark, opposite five-lobed leaves, and horizontally spreading winged fruits. It is mainly found in sparse forests at elevations of 500–1200 m in Zhejiang, northern Fujian, southern Anhui, and eastern Jiangxi [19,20,21]. It is a valuable hardwood because of its straight grain, hard texture, fine structure, and erosion-resisting heartwood. The species is also a high-quality ornamental and landscaping tree because of its elegant shape and golden-yellow foliage in autumn, possessing significant ecological and economic value [22]. Although previous studies have modeled the suitable habitats of closely related species such as *A. truncatum*, *A. cordatum*, and *A. poliophyllum* [23,24,25], there is still a lack of systematic research on the resource distribution and suitable habitats of *A. pubinerve*. In May 2023, we conducted a native plant survey on the Xishan Island in Suzhou, Jiangsu province, and discovered a wild population of *A. pubinerve* Rehder near Tianwangwu village (Figure 1). We determined the species based on careful examination of morphological characters, type specimen (*E. Faber* 203) and the relevant literature [26]. This represents the first confirmed record of *A. pubinerve* in Jiangsu and also constitutes the northernmost known occurrence (31.12° N, 120.28° E). This finding extends the known range of the species in the north, because neither the Flora of Jiangsu nor the ‘Catalogue of Life China 2024 Annual Checklist’ included Jiangsu in the statement of its distribution [27]. It is interesting to know how this new distribution record impacts on our knowledge of the species’ ecological niche and potential habitat under climate change.

This study aims to systematically analyze the distribution pattern and climate responsing mechanisms of *A. pubinerve* by integrating multi-source data and model optimization methods. The specific objectives include: (1) using the MaxEnt model optimized by the ‘ENMeval’ package to simulate the dynamic changes of suitable habitats under current and three future climate scenarios (SSP126, SSP370, and SSP585); (2) to identify the main environmental factors affecting the species’ distribution; (3) based on the simulation results, to provide scientific basis for the conservation and rational utilization of the species’ resources. This study is expected to provide new data support for the fundamental research of *A. pubinerve* and to offer a theoretical basis for related conservation practices and resource utilization decisions. The newly discovered distribution record from the Xishan Island of Suzhou during the research process also provides important clues for further understanding the species’ distribution range.

## 2. Results

### 2.1. Model Optimization and Precision Evaluation

**Figure 2:**
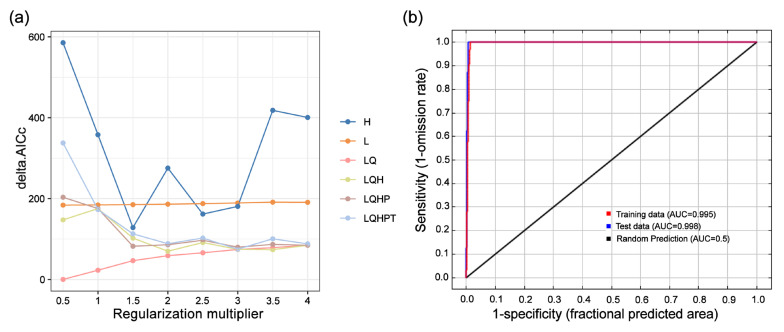
Model evaluation results (Delta.AICc and ROC curve) for *Acer pubinerve*: (**a**) Delta.AICc values corresponding to various combinations of feature classes and regularization parameters in the MaxEnt model; (**b**) ROC curve showing the performance of the MaxEnt model for *Acer pubinerve*. Note: the legend represents the feature class types-L: Linear, Q: Quadratic, H: Hinge, P: Product, and T: Threshold.

The MaxEnt model parameters were optimized using the ‘ENMeval’ package. The combination of RM = 0.5 and feature class LQ yielded the lowest AICc value, indicating the lowest model complexity and reduced risk of overfitting based on the Akaike Information Criterion. Therefore, this parameter set was selected as the optimal configuration for the model (Figure 2a). According to the ROC curve results (Figure 2b), the AUC values of the training and test datasets were 0.995 and 0.998, respectively, both approaching the theoretical maximum value.

### 2.2. Dominant Environmental Factors Impacting the Distribution of Acer pubinerve

We performed contribution rate and permutation importance analysis of environmental factors affecting the distribution of suitable habitats for *A. pubinerve* based on the ‘Jackknife test’ in the MaxEnt model (Table 1). Among the eight environmental variables included in the simulation, temperature factors had the highest overall contribution rate, reaching 75.1%. Among them, the contribution of the mean temperature of the warmest quarter (bio_10) was the most significant, while the mean temperature of the wettest quarter (bio_8) had the lowest contribution rate. The total contribution rate of precipitation factors was 24.8%, with annual precipitation (bio_12) having the highest contribution among them, and precipitation seasonality (bio_15) having the lowest. The mean temperature of the driest quarter (54.9%) and mean temperature of the wettest quarter (27.4%) all had relatively high permutation importance values (Table 1). The results indicated that the mean temperature of the warmest quarter, the mean temperature of the driest quarter, and annual precipitation were the main environmental factors affecting the habitat suitability of *A. pubinerve*.

### 2.3. Current Distribution Pattern and Suitable Habitats

**Figure 3:**
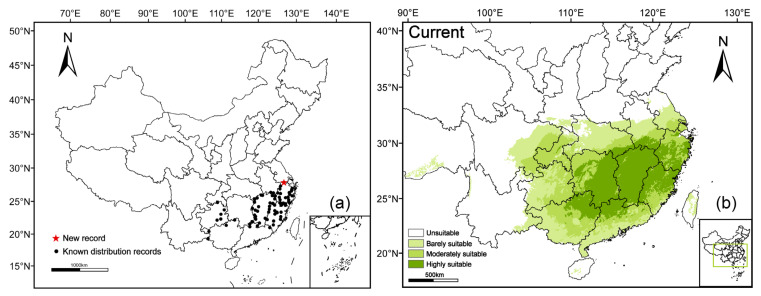
Distribution status and potentially suitable areas for *Acer pubinerve* in the current study. (**a**) *Acer pubinerve* distribution occurrences; (**b**) current potentially suitable areas for *Acer pubinerve*; Base map review number: GS (2020)4619.

The current distribution points and predicted suitable distribution map of *A. pubinerve* were generated using ArcGIS 10.8. *Acer pubinerve* was most widely distributed in Zhejiang, followed by Jiangxi and Fujian provinces (Figure 3a). Additionally, the predicted range covering the known distribution points of *A. pubinerve* was consistent with the actual distribution (Figure 3b).

**Figure 4:**
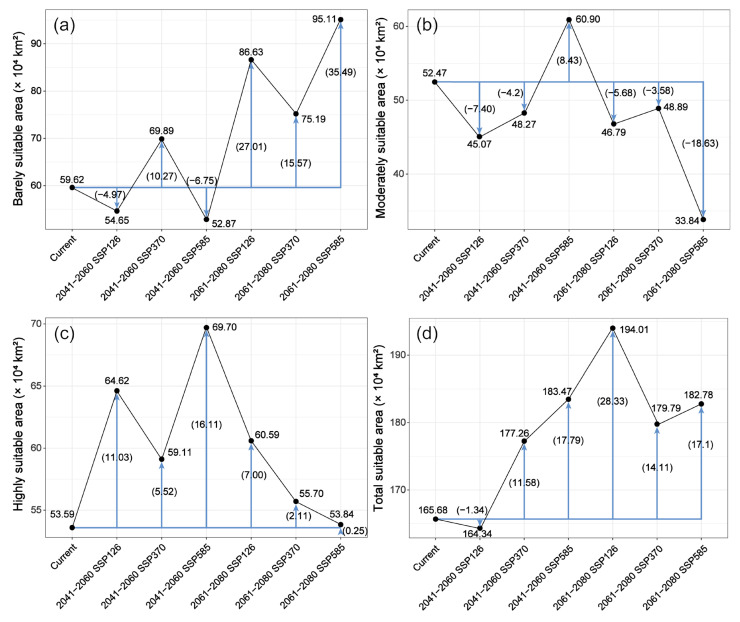
The suitable area of *Acer pubinerve* under current and future climate scenarios. (**a**) Barely suitable area; (**b**) Moderately suitable area; (**c**) Highly suitable area; (**d**) Total suitable area. Black lines show absolute area changes across time periods and SSPs (×10^4^ km^2^); blue arrows indicate changes relative to the current period, with values in brackets.

According to the prediction results of the MaxEnt model under the current climate scenario, *A. pubinerve* had a widespread potential habitat in Zhejiang, Fujian, Jiangxi, Guizhou, Guangdong, Guangxi, Hunan, Hubei, Chongqing, Shanghai, as well as parts of Anhui, Jiangsu, Sichuan, Henan, Shanxi, Yunnan, Xizang, Taiwan, and Hainan (Figure 3b). The total suitable habitat area (*p* ≥ 0.01) of the species under the current climate amounted to 165.68 × 10^4^ km^2^. The highly suitable area (*p* ≥ 0.5) was mainly distributed throughout the Jiangxi province, as well as in northern parts of Fujian, Guangxi, and Guangdong, and in southern parts of Zhejiang, Hubei, and Anhui, as well as eastern Hunan, covering an area of 53.59 × 10^4^ km^2^, and accounting for approximately 32.35% of the total suitable habitat area. The moderately suitable area (0.3 ≤ *p* < 0.5) was distributed in the whole of Chongqing and Shanghai, southern Jiangsu and Fujian, central Guangdong and Guangxi, eastern Guizhou, Sichuan, and Hubei, western Hunan, and southeastern Anhui. The area amounted to 52.47 × 10^4^ km^2^, accounting for 31.67% of the total suitable habitat area. The barely suitabile area (0.1 ≤ *p* < 0.3) was distributed in eastern Sichuan and southern Guangdong, Guangxi, Shaanxi, and Henan, southeastern Yunnan and Xizang, north–central Hubei, and central parts of Anhui, Jiangsu, Guizhou, Hainan, and Taiwan. The area covered 59.62 × 10^4^ km^2^, accounting for 35.99% of the total suitable habitat area (Figure 4).

### 2.4. Distribution of Suitable Habitat Under Different Future Climate Scenarios

**Figure 5:**
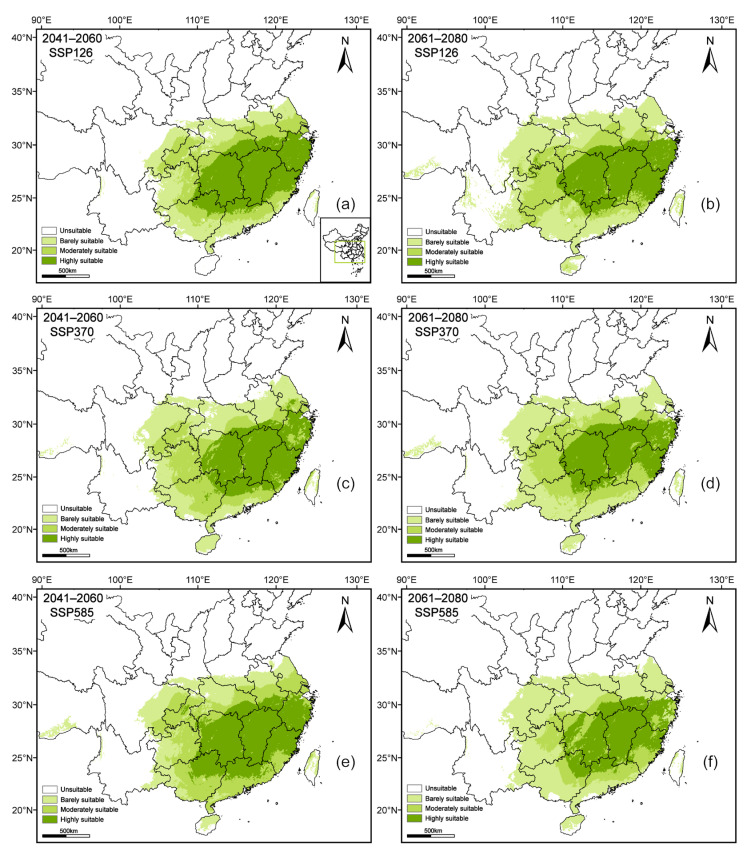
The predicted areas for *Acer pubinerve* under different climate conditions. Note: different letters indicate different future climate scenarios: (**a**) SSP126 2041–2060; (**b**) SSP126 2061–2080; (**c**) SSP370 2041–2060; (**d**) SSP370 2061–2080; (**e**) SSP585 2041–2060; (**f**) SSP585 2061–2080.

Based on the optimized MaxEnt model predictions, the suitable habitat area and spatial distribution of *A. pubinerve* would undergo significant changes under different future climate scenarios (Figure 4 and Figure 5). In terms of changes in suitable habitat area (Figure 4), compared with the current climate conditions, the total suitable habitat area showed a trend of first decreasing and then increasing under all future scenarios, with the largest increase occurring under the SSP126 scenario during 2061–2080. The highly suitable habitat area would significantly expand under the SSP585 scenario during 2041–2060 and would reach its maximum value. The area of the moderately suitable habitat showed a decreasing trend under most scenarios, with the most significant decline occurring under the SSP585 scenario during 2061–2080, reaching its minimum value. In contrast, the area of the barely suitable habitat would increase in the future and reach its maximum under the SSP585 scenario during 2061–2080.

The spatial distribution maps further indicated that under future climate change, the suitable habitats would remain primarily concentrated in provinces such as Jiangxi, Zhejiang, Fujian, and Hunan, with highly suitable areas showing a tendency to expand northward and westward (Figure 5). In addition, under the SSP585 scenario during 2061–2080, the highly suitable habitats exhibited a trend of fragmentation, with a reduction in moderately to highly suitable areas and an increase in the proportion of barely suitable areas.

### 2.5. The Spatial Changes and Centroid Migration of the Suitable Habitat of Acer pubinerve

**Figure 6:**
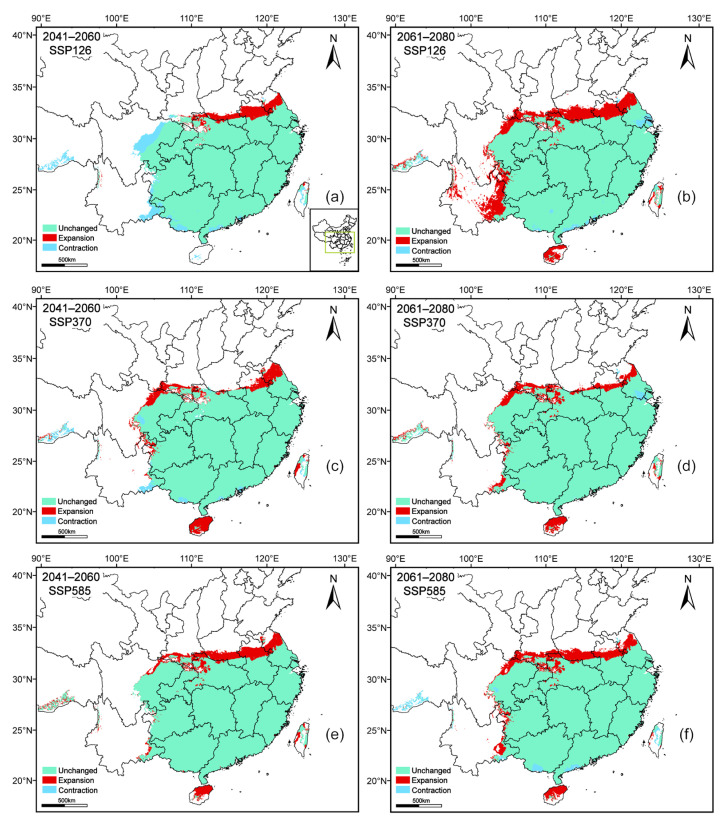
Spatial dynamics of *Acer pubinerve* under different future climate scenarios. Note: different letters indicate different future climate scenarios: (**a**) SSP126 2041–2060; (**b**) SSP126 2061–2080; (**c**) SSP370 2041–2060; (**d**) SSP370 2061–2080; (**e**) SSP585 2041–2060; (**f**) SSP585 2061–2080.

To assess the impact of climate change on *A. pubinerve* under different conditions, we predicted the areas of habitat expansion, stability, and contraction by comparing its current and future distributions (Figure 6). The results indicated that the species exhibited a clear overall trend of northward expansion, with the expansion areas mainly concentrated along the northern edge of its current distribution. Meanwhile, the contraction areas were relatively limited, primarily located at the western margin and in southeastern Xizang.

**Figure 7:**
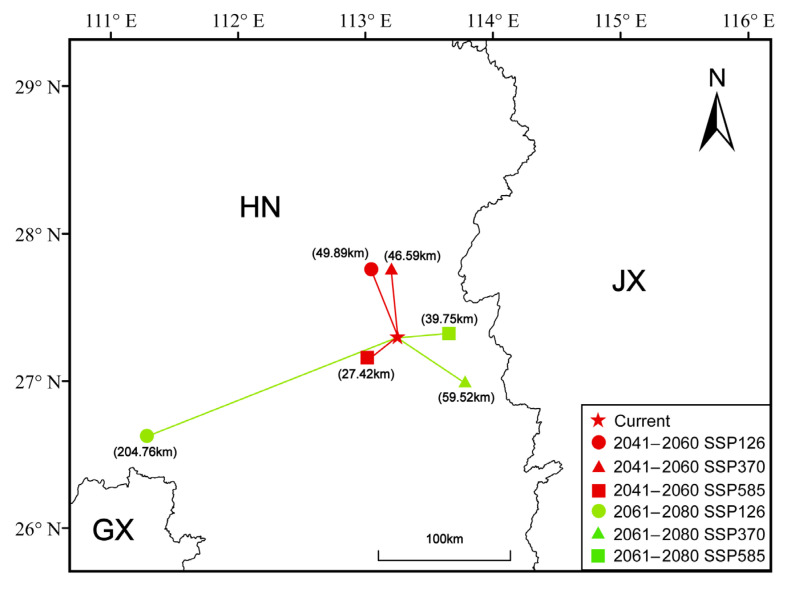
Centroid migration of *Acer pubinerve* under future climate change scenarios. HN: Hunan province; GX: Guangxi province; JX: Jiangxi province.

We assessed the spatial distribution under future climate scenarios by computing the centroid of suitable habitats in each case. The results showed a trend of a westward shift in the centroid under all scenarios, except for SSP370 and SSP585 during 2061–2080. Among them, the greatest migration distance was observed under the SSP126 climate scenario during 2061–2080, while the shortest migration distance occurred under the SSP585 climate scenario during 2041–2060 (Figure 7).

## 3. Discussion

### 3.1. Optimization and Evaluation of the MaxEnt Model

Climate change significantly affects plant survival and distribution patterns, making it a focal area of research in plant ecology [28,29,30]. In this study, the MaxEnt model was used to evaluate the potential suitable habitats of *A. pubinerve*. By analyzing the relationship between known species occurrence points and environmental variables, this model can not only identify suitable habitats under current climatic conditions but also simulate potential distributions under historical and future climate scenarios [31]. Although MaxEnt performs robustly in complex environmental settings and large-scale spatial analyses, directly using default parameters may lead to model over-complexity and an increased risk of overfitting [5].

To improve prediction accuracy, this study systematically evaluated forty-eight parameter combinations (eight regularization multipliers × six feature combinations) using the ‘ENMeval’ package. The results show that the combination of RM = 0.5 and FC = LQ is optimal, reducing the delta.AICc value from 172.70 under the default parameters to 0, which significantly decreases model complexity and the tendency toward overfitting. In addition, the high AUC value and the results of the Jackknife test both confirmed the reliability of the model’s predictive accuracy. Similar optimization strategies have been successfully applied in distribution prediction studies of species such as *Acer negundo* and *Juniperus pseudosabina* [32], indicating that parameter tuning has general significance for enhancing the ecological application value of the MaxEnt model. This approach is particularly suitable for species conservation and management, as accurate distribution prediction provides a scientific basis for formulating effective conservation measures.

### 3.2. Potential Geographical Distribution and Influencing Climate Factors of Acer pubinerve

Climate conditions significantly influence the growth and development of organisms and the spatial patterns of biodiversity [33]. Among various climatic variables, moisture and temperature are key driving forces that jointly shape the geographical distribution patterns of plants [34]. However, their influence varies significantly across different species [35]. In this study, we found that the spatial distribution pattern of *A. pubinerve* is jointly determined by precipitation and temperature. We determined that mean temperature of warmest quarter, mean temperature of driest quarter, and annual precipitation are the main environmental factors influencing the distribution of *A. pubinerve*. The contribution rate of temperature variables is significantly higher than that of precipitation variables, implying that temperature has a stronger impact on the distribution of this species (Table 1). This result is consistent with findings from studies on *Acer* species in subtropical regions, such as *Acer truncatum*, *Acer calcaratum*, and *Acer oligocarpu*, confirming the dominant role of temperature in determining the distribution of *Acer* species [25,36]. Another study on woody dicotyledonous plants in humid regions of China indicates that annual precipitation has a significant impact on plant distribution patterns [37], which further supports our findings.

### 3.3. Potential Geographic Distribution of Acer pubinerve Under Future Climate Change Scenarios

The impact of climate change on the distribution of plant suitable habitats shows significant species specificity [38]. Previous studies have shown that under global warming, cold-tolerant species from northern regions often face the dual threats of habitat deterioration and genetic diversity loss, while thermophilous species may expand their distribution range [38,39,40]. Our study confirms this conclusion. The simulation results show that, compared with current climatic conditions, the potential suitable habitat of *A. pubinerve* generally exhibits an expansion trend (except under the SSP126 scenario for 2041–2060), which is consistent with the findings of *Acer truncatum* (Figure 4) [36]. Spatial analysis indicates that the expansion of suitable habitats primarily occurs along the northern margin of the current distribution area, which is consistent with the newly recorded northernmost occurrence of our field investigation. This suggests that with ongoing climate warming, the suitable range of *A. pubinerve* may gradually shift northward. In contrast, contraction areas are mainly concentrated along the western margin and southeastern Xizang (Figure 6). Due to the greater extent of expansion compared to contraction, the suitable habitat exhibits a net increasing trend, a phenomenon consistent with findings from studies on several temperature-sensitive plant species [41,42]. This expansion may result from climate change optimizing hydrothermal conditions, thereby providing a more favorable growth environment for *A. pubinerve* and enhancing its cultivation potential in newly suitable areas [43,44,45].

The analysis of population centroid movement provides an effective indicator for predicting changes of species’ distribution. Previous studies have shown that, at the global or regional scale, the distribution center of most species tends to undergo long-distance migration [46]. This study found that the distribution centroid of *A. pubinerve* generally exhibits a westward migration trend (except under the SSP126 and SSP370 scenarios for 2061–2080), a pattern similar to the distributional shift observed in *Cryptomeria fortunei* [47]. Under different climate scenarios, there are certain variations in the direction of centroid migration. This uncertainty probably arises from the intensity of human activity interference and the spatiotemporal heterogeneity of global warming [47]. It is noteworthy that the centroid migration distance of *A. pubinerve* varies significantly under future scenarios, with the most pronounced displacement occurring under the SSP585 scenario for 2061–2080. Nevertheless, the overall centroid of the suitable habitat remains relatively stable, primarily located within the Hunan province, indicating that the core distribution area of this species exhibits strong adaptability to climate change.

### 3.4. Conservation and Utilization of Acer pubinerve

Our prediction results based on the MaxEnt model show that the suitable distribution range of *A. pubinerve* in China is relatively wide, covering not only the documented distribution areas such as Anhui [48,49,50], Fujian [51], Guangdong [52,53], Guangxi [54,55], Guizhou [56], Jiangxi [57,58] and Zhejiang [59,60], but also extending to Jiangsu, Hunan, Hubei, Sichuan, Chongqing, Shanxi, Xizang, Hainan, and Taiwan. Although the potential distribution range predicted by the model is broad, the realized distribution of the species remains limited in comparison, i.e., the species is present in only a portion of the areas that are climatically suitable. This discrepancy is likely due to the following factors. First, seedlings of *A. pubinerve* have a weak adaptability to shaded environments. High forest canopy density can limit growth and increase mortality rates of seedlings, thus negatively impacting population recruitment. Nevertheless, this constraint does not uniformly affect all regions; in many areas within the current range, *A. pubinerve* establishes itself successfully in open or disturbed habitats where light conditions are favorable [61,62]. Secondly, interspecific competition limits the dominance of *A. pubinerve* within local communities. Finally, habitat loss and over-exploitation including logging are leading to the loss of high-quality parent tree resources and genetic diversity, further impacting the actual distribution [22]. As a result, we suggest taking effective measures to conserve the resources of the species, e.g., in situ conservation to protect the living environment and enhance population size, and ex situ conservation—particularly in regions where suitable habitats are expected to shrink under future environmental conditions—to conduct artificial propagation and reintroduce the species to its natural distribution areas. It is also necessary to conduct basic biological research of the species to provide scientific supports for both conservation and utilization purposes.

### 3.5. Problems and Future Directions

It is important to note that species’ distribution is often jointly shaped by multiple environmental factors. Studies have shown that, in addition to climatic factors, abiotic factors such as topography and soil also play significant roles in species’ distribution. For example, by integrating 601 distribution points of *Eucommia ulmoides* and 24 environmental variables, Liu et al. suggested that topographic factors, along with temperature and precipitation, jointly influenced the suitable distribution of *E. ulmoides* [63]. Similarly, Li et al. predicted the suitable habitat of *Pinus massoniana* based on 294 distribution records and 18 environmental variables, and concluded that soil factors as the key environmental variables determined the distribution of *P. massoniana* [64]. These findings indicate that topographic and soil factors have significant impacts on the distribution of tree species and may also impact the distribution of *A. pubinerve*. Moreover, previous studies have demonstrated that geographical factors such as slope, aspect, and elevation, as well as biotic factors including human disturbances, interspecific competition, and the species’ biological characteristics, have contributed to the spatial heterogeneity of species distribution [43]. Therefore, we recommend incorporating more multidimensional environmental variables, high-resolution data, and targeted field investigations in future studies to validate and improve the model in order to better understand the underlying mechanisms of species distribution. Notably, in this study, the newly recorded population of *A. pubinerve* in the Jiangsu province provides empirical support for the model’s prediction of suitable areas expanding northward under climate warming. This cross-validation between field discovery and modeling results highlights the importance of integrating species distribution modeling with field surveys to more accurately assess species range dynamics.

## 4. Materials and Methods

### 4.1. Species Distribution Data Preparation

We collected species distribution data mainly from the following sources: (1) our field surveys in Suzhou of the Jiangsu province during July 2021 and May 2023; (2) open databases including ‘Plants of the World Online’ (POWO, https://powo.science.kew.org, accessed on 13 March 2024), ‘Chinese Virtual Herbarium’ (CVH, http:// www.cvh.ac.cn, accessed on 12 March 2024), ‘Global Biodiversity Information Facility’ (GBIF, http://www.gbif.org, accessed on 12 March 2024) [65], ‘Catalogue of Life China 2024 Annual Checklist’, ‘Flora of China’; (3) the published literature. We then screened the preliminary distribution data and removed anomalous distribution points, introductory cultivation distribution points, and duplicate distribution data. For records with locality information but lacking coordinates, we used Google Earth (http://www.google.cn, accessed on 15 March 2024) to obtain coordinates. In this study, a spatial filtering method was applied to optimize the species distribution data by retaining only one occurrence record within each 1 km × 1 km grid cell [7]. This approach effectively reduced sampling bias and spatial autocorrelation effects, significantly improving the spatial representativeness of the dataset and the reliability of the analytical results. Finally, we assembled a dataset including 73 valid occurrence records of *A. pubinerve* (Table S1).

### 4.2. Environmental Data Acquisition and Screening

We obtained 19 environmental variables under current and future climate scenarios (Table 1) from the Global Climate Database (WorldClim, http://www.worldclim.org, accessed on 10 May 2025) at a spatial resolution of 30 arc-seconds (~1 km^2^). Two time periods, 2041–2060 and 2061–2080, were included. The future climate scenarios were selected from three representative concentration pathways under the BCC-CSM2-MR model in the new emission scenarios released by the Intergovernmental Panel on Climate Change (IPCC) in 2021: SSP126, SSP370, and SSP585 [11]. Among them, SSP126 represented a sustainable development pathway with low greenhouse gas emission concentrations. SSP245 represented a moderate development pathway with medium greenhouse gas emission concentrations. SSP585 represented a development pathway primarily based on fossil fuel use, with high greenhouse gas emission concentrations. The three climate change scenarios represented optimistic, moderate, and pessimistic scenarios, respectively [66].

To reduce the multicollinearity effects between environmental factors, prevent overfitting in the model, and enhance the reliability of the prediction results [67,68], we conducted a preliminary simulation experiment for *A. pubinerve*. Then, ArcGIS software was used to extract the values of environmental factors at species distribution points, and the correlations between variables were assessed using ‘Variance Inflation Factors’(VIF) and ‘Pearson’ correlation analysis methods. The correlation coefficients of the environmental variables were calculated using the ‘cor’ function in R, and the VIF was computed using the ‘usdm’ package [5,69]. By integrating the results of the preliminary simulation experiment with the correlation analysis data, climate factors with a correlation coefficient r < |0.7| and VIF < 10 were selected. Ultimately, eight environmental variables were determined for the suitable habitat prediction of *A. pubinerve*.

### 4.3. MaxEnt Model Construction and Evaluation

In this study, the ‘ENMeval’ package in R was used to optimize the parameters of the MaxEnt model. For the setting of the regularization multiplier (RM), a range of values from 0.5 to 4 was selected, with an interval of 0.5, resulting in a total of eight different parameter values tested. Regarding feature combinations (FC), based on the feature types provided by the MaxEnt model—linear (L), quadratic (Q), hinge (H), product (P), and threshold (T)—six combinations were evaluated: L, LQ, H, LQH, LQHP, and LQHPT [5]. The ‘ENMeval’ package was used to systematically test the above 48 parameter combinations (8 RM values × 6 FC types), and the corrected Akaike Information Criterion (AICc) was applied to evaluate the goodness of fit and complexity of the models. Ultimately, the parameter combination with the lowest delta. The AICc value was selected as the optimal model configuration.

Subsequently, based on the optimized model parameters, we applied MaxEnt 3.4 and ArcGIS 10.8 software platforms to predict and analyze the potential suitable habitats of *A. pubinerve* under different climate scenarios. For the species modeling, the sample data was split into training and test sets in a 75% to 25% rate, with 10,000 background points, and the simulation was repeated 20 times to improve the reliability of the results. To assess the importance of each bioclimatic variable, we used the Jackknife test to calculate their contribution ratio. The model accuracy was evaluated using the Receiver Operating Characteristic (ROC) curve, and the Area Under the Curve (AUC) value. The AUC ranges between 0 and 1, with higher values indicating stronger discriminative ability of the model. Generally, an AUC greater than 0.8 is considered to reflect high predictive accuracy and relatively good model performance [47,70].

### 4.4. Visualization and Spatial Analysis of Suitable Habitats

Based on the simulation results of the MaxEnt model, raster data processing and reclassification were conducted using ArcGIS 10.8 software. According to the natural breaks classification method, the potential habitat of *A. pubinerve* was divided into four suitability levels: unsuitable area (*p* < 0.1), barely suitable area (0.1 ≤ *p* < 0.3), moderately suitable area (0.3 ≤ *p* < 0.5), and highly suitable area (*p* ≥ 0.5). These classifications were visualized, and the area of each suitability zone was calculated.

To investigate the impact of climate change on the distribution of suitable habitats, a dual analysis approach was adopted in this study. First, the ‘Quick Reclassify to Binary’ function of SDMtools was used to convert ASC files from different periods into binary data, using 0.1 as the threshold (presence probability ≥0.1 was assigned a value of 1, and <0.1 was assigned a value of 0) [7]. Subsequently, the ‘Distribution Change Between Binary SDMs’ tool in the SDMtools 2.5 plugin was used on the ArcGIS 10.8 platform to compare distributions across different periods with the current distribution, thereby identifying stable areas, expansion areas, and contraction areas. In addition, we quantitatively analyzed the spatial evolution characteristics of habitat distribution by calculating the spatial centroid of suitable habitats in each period. Based on the distance between the centroids under future climate scenarios and the current centroid, the magnitude of spatial displacement of suitable habitats was further quantified, thereby revealing the dynamic patterns of potential distribution of *A. pubinerve* under climate change.

## 5. Conclusions

This study provides the first comprehensive assessment of the distribution and dynamic shifts of suitable habitats of *Acer pubinerve* under current and future climate change scenarios. Temperature is the main limiting climatic factor for the distribution of the species, followed by precipitation. Under the current climate condition, the suitable habitats of *A. pubinerve* are widely distributed across provinces including Zhejiang, Fujian, Jiangxi, Guizhou, Guangdong, Guangxi, Hunan, Hubei, and Chongqing, and also extend to certain areas of Anhui, Jiangsu, Sichuan, Henan, Taiwan, Shanxi, Xizang, and Hainan. Under different climate change scenarios, the total area and geographic range of suitable habitats for *A. pubinerve* are projected to expand overall, with relatively high distributional stability observed in Zhejiang, Fujian, Jiangxi, and Hunan. Our study clarifies the key environmental factors influencing the distribution of *A. pubinerve*, which provides an important reference for breeding, conservation, and landscaping plantation of this species under different climate change scenarios. Importantly, the new record of *A. pubinerve* in the Jiangsu province represents the northernmost boundary of its known range and corresponds well with the northern expansion trend predicted by the MaxEnt model, further validating the model’s reliability and demonstrating the value of integrating field surveys with ecological modeling.

## Figures and Tables

**Table 1:** Contribution rate and permutation importance value of each environmental variable.

Variables	Description	Units	Percent Contribution (%)	Permutation Importance	Correlation	VIF
bio_1	Annual mean temperature	°C	–	–	–	–
bio_2	Mean diurnal range	°C	12.6	0.1	0.4	4.4
bio_3	Isothermality	–	11.5	9.9	0.5	6.2
bio_4	Temperature seasonality	°C	–	–	–	–
bio_5	Max temperature of warmest month	°C	–	–	–	–
bio_6	Min temperature of coldest month	°C	–	–	–	–
bio_7	Temperature annual range	°C	–	–	–	–
bio_8	Mean temperature of wettest quarter	°C	1.6	27.4	0.4	3.0
bio_9	Mean temperature of driest quarter	°C	24.6	54.9	0.5	4.4
bio_10	Mean temperature of warmest quarter	°C	24.8	0.4	0.4	4.1
bio_11	Mean temperature of coldest quarter	°C	–	–	–	–
bio_12	Annual precipitation	mm	18.2	0.6	0.4	5.1
bio_13	Precipitation of wettest month	mm	–	–	–	–
bio_14	Precipitation of driest month	mm	–	–	–	–
bio_15	Precipitation seasonality	–	1.9	6.6	0.4	3.3
bio_16	Precipitation of wettest quarter	mm	–	–	–	–
bio_17	Precipitation of driest quarter	mm	–	–	–	–
bio_18	Precipitation of warmest quarter	mm	4.7	0	0.4	4.1
bio_19	Precipitation of coldest quarter	mm	–	–	–	–

## Data Availability

All data used in the study are included in this paper.

## Supplementary Materials

The following supporting information can be downloaded at: https://www.mdpi.com/article/10.3390/plants14131895/s1, Table S1: Distribution data for *Acer pubinerve*.
